# Cancer of the Uterus Treated by "Rays"

**Published:** 1903-01-17

**Authors:** 


### CANCER OF THE UTERUS TREATED BY "RAYS."

THE treatment of malignant disease by "rays" of various kinds is being so largely employed that we need hardly be surprised to hear of its application to the uterus, although the technical difficulties to be overcome in reaching disease in such a situation must be very considerable. Dr. Margaret A. Cleaves[1] gives a very full report of a case of inoperable cancer of the cervix uteri and adjacent tissues which was treated by the $x$-rays and chemical light rays. The patient was 42 years of age. On physical examination it was found that the cervix uteri was one mass of cauliflower excrescence, which crumbled off at touch and bled profusely, and that both broad ligaments were infiltrated, more especially the left one. The uterus was somewhat enlarged and immobile from pelvic infiltration. There was characteristic dirty and ill-smelling discharge, and perforation of the recto-vaginal septum seemed imminent. On consultation it was decided that operation was out of the question. Treatment by electricity, light, and "rays" was thereupon commenced. Into the details we need not now enter, beyond saying that they included the whole gamut of electro-physical means, the hydro-electric douche, the convective discharge of the Franklinic current, the arc-light bath, the $x$-ray treatment, and the ultra-violet rays. After five months and one week, during which time 110 "treatments" were given, physical examination showed that the tissues of the vagina had become soft and non-resistent; the cervix was of irregular contour, but was covered with normal mucous membrane, except at a point which had been burned by a touch of the anode; the cul-de-sac was free on the right side, but on the left the fundus was drawn over by scar tissue; there were no enlarged glands, nor evidence of pelvic infiltration; the os was patulous, and there were no nodules in the broad ligaments. The case is not reported as a cure, but to show what has been done in a desperate condition, and to call attention to the value of the $x$-rays and ultra-violet light in combination for this class of cases.

[1] New York Med. Rec., Dec. 13.

